# Organ transplantation and meaning of life: the quest for self fulfilment

**DOI:** 10.1007/s11019-012-9439-z

**Published:** 2012-09-27

**Authors:** Jacques Quintin

**Affiliations:** Université de Sherbrooke, 97 Heneker, Sherbrooke, QC J1J 3G2 Canada

**Keywords:** Nature, Naturalism, Phenomenology, Hermeneutics, Aristotle, Descartes, Meaning, Wisdom, Organ transplantation

## Abstract

Today, the frequency and the rate of success resulting from advances in medicine have made organ transplantations an everyday occurrence. Still, organ transplantations and donations modify the subjective experience of human beings as regards the image they have of themselves, of body, of life and of death. If the concern of the quality of life and the survival of the patients is a completely human phenomenon, the fact remains that the possibility of organ transplantation and its justification depend a great deal on the culture in which we live. The exploration of the philosophical tradition allows for a reconsideration of organ transplantation. If we listen to people who have experienced the decline of one of their organs and their own rebirth through the organ of someone else, we arrive at the conclusion that they went through an extreme experience in which nothing appeared as before. All those experiences intensify philosophical questionings on the meaning of life with respect to self fulfilment. The concept of nature as the experience of others can be an authentic source from which to nourish our thoughts about organ transplantation. However, and this is our hypothesis, we need something more if we are to decide something about our own life. We need a hermeneutical stance in relation to ourselves and to our world. Philosophical counselling, as a long established tradition originating with Pythagoras and later reframed by the German philosopher Achenbach could be useful in inspiring a reflection on the good life, chiefly as it takes the form of a Socratic dialogue.

## Introduction

The middle of the last century saw the first successful attempts at kidney transplantations. Other successful transplantation experiments soon followed: liver, heart, lungs and pancreas. Today, the frequency and the rate of success resulting from advances in medicine have made organ transplantations an everyday occurrence (Linden 2009). Still, organ transplantations and donations modify the subjective experience of human beings as regards the image they have of themselves, of body, of life and of death. The blending of bodies also challenges formerly indisputable natural limits. Furthermore, transplantation eventually leads to a questioning of the very purpose of medicine: does it exist to care, to cure and to relieve suffering or does it exist to improve the human race?

Organ transplantation thus poses a certain number of inescapable ethical problems. Those who become involved in the sphere of organ transplantation raise the question of consent, the definition of death, the notion of mutilation of the body, the rules of distribution of organs, or the transformation of the body into a business affair. Hence, ethicists are generally asked to remedy these problematical situations by bringing forth normative or prescriptive rules as though ethics could be reduced to resolving problems by eschewing the question of the meaning of life, i.e. the question of fulfilment of the self. The risk here consists in managing such a situation by brushing aside philosophical questions, such as those regarding the meaning of life and death, which are appropriate questions on human existence and what constitutes the good life. This reflection on the good life may become a part of a greater quest for wisdom that is often grounded in the knowledge of reality, i.e. what must be is prescribed by nature. In this sense, wisdom has long been associated with harmony as established by nature, except that this wisdom appears differently to the ancient Greeks, to the Moderns and to contemporaries. They each have their own way of deliberating on the good life.

If the concern of the quality of life and the survival of the patients is a completely human phenomenon, the fact remains that the possibility of organ transplantation and its justification depend a great deal on the culture in which we live. The exploration of the philosophic tradition allows reconsidering otherwise the organ transplantation as a simple replacement of exchangeable body parts. Thus, the concept of nature, as elaborated by Aristotle and Descartes and its representation within naturalism may be used to show how organ transplantation is or is not justified. Then, an examination of a phenomenological and hermeneutical perspective will allow us to sidestep the stumbling block of essentialism. If we listen to people who have experienced the decline of one of their organs and their own rebirth through the organ of someone else, we arrive at the conclusion that they went through an extreme experience in which nothing appeared as before: that of being one person and multiple people at the same time, of having within them a living dead person, of having their own life through the death of another, of being a survivor. All those experiences intensify the philosophical questionings on identity, on the relationship between soul and body, on the ontological status of the body, and principally on the meaning of life.

The concept of nature as the experience of others all can be an authentic source to nourish our thought about organ transplantation. But, and this is our hypotheses, we need something more if we want to decide something about our life. We need a hermeneutical stance about oneself and our world. Philosophical counselling, as a long tradition from Pythagoras and so on, reframed by the German philosopher Gerd Achenbach in the 1980 could be helpful to reflect on the good life, mainly if it takes the form of a Socratic dialogue.

## The division between facts and values

Since the time of Plato, one of the characteristics of philosophy has been to provide justification. The question then consists in knowing how to justify organ transplantation within the meaning of human existence. This consideration of the question of meaning widely exceeds modern science’s empirical analysis of the causes. We are, therefore, confronted with the split between the power of instrumental rationality and the wisdom derived from axiological rationality, as well as with the confusion between the legitimacy of scientific knowledge and the legitimacy of its common practice (Wolff 2010, p. 19).

This division was first underlined by David Hume in his *Treatise of the Human Nature* published in 1739. He observed that our arguments often proceed from a description of facts which ends in a normative claim. For Hume, this kind of leap was unacceptable. G.E. Moore, in his *Pincipia Ethica* published in 1903, took up this problem by elaborating the concept of “naturalistic fallacy”, albeit by approaching it from the opposite direction.

For Moore, it was wrong to analyze an ethical proposition by defining the good by way of natural properties. For some, the world of facts described by science and the world of values described by ethics must remain different. With the advent of positivism, science even made it a point of honour not consider values as possible objects of science. However, the development of the cognitive sciences (Gardner 1985; Dupuy 1994; Ganascia 1996) integrated within the paradigm of naturalism, raises the taboo surrounding the division between facts and values. Values then appear as epiphenomena of nature.

## The point of view of Aristotle

From a hypothetical perspective, even if the ancient Greeks had acquired the necessary knowledge and technical means to perform organ transplantation, it is highly unlikely that they would have accepted the practice, as they had no conceptual framework that would allow them to justify such a procedure. What would such a conceptual framework require? For practical reasons, let us limit ourselves to that of Aristotle.

In his writings, Aristotle often refers to medicine. It appears as a model by which to consider nature, ethics and politics. This comes as no surprise when we consider that his father was a doctor in the court of the king of Macedonia. It is quite probable that Aristotle was schooled directly by his father in the rudiments of the biology and medicine of the age. Although Aristotle showed an interest in medicine, it was mainly biology and anatomy that held his attention and inspired his quest to better understand nature.

Aristotle’s major contribution to ancient medicine consists in his doctrine of the four fundamental qualities, that is to say, warmth, cold, wet and dry. It is these four fundamental qualities that make possible the notion of harmony and homeostasis in ancient medicine and contribute to the health of the body. Therefore, Aristotle placed knowledge of nature above medicine. Even today, medical students begin their studies by learning the fundamental sciences before integrating the study of the various systems, such as the endocrine system, the nervous system, the circulatory system. Only at the end of their medical training do they study the clinical aspect.

How exactly did Aristotle view nature? In *Physics*, Aristotle indicated that “of things that exist, some exist by nature, some from other causes” (1941, p. 236). Things that exist according to nature find in themselves their own cause. “Each of them has within itself a principle of motion and of immobility” (1941, p. 236). This is how Aristotle defined nature. It is the principle and the cause of movement and of rest of things by design and not by accident (1941, p. 251). In short, nature is an immanent principle of spontaneity: nature acts as a man who cures himself (1941, p. 251). This is why health is a reflection of the balance of the elements of nature. This balance cannot be imposed from without, by accident.

Nature is the shape that is outlined in the definition of the thing (1941, p. 237), and this shape does not exist separately from the thing, “except in statement” (1941, p. 238). Aristotle, in his *Parts of Animals*, warns readers that what is under discussion is not the material part, “but the relation of such part to the total form” (1941, p. 657). In this way, for human beings, the body represents the material, and the soul, the form that give the body its constitution and its principle of movement. Thus, “soul is the actuality of a body” (1941, p. 555). However, if the soul is common to all living things, these faculties are not distributed in equal measure. Plants have only the nutritional faculty, while animals possess, in addition, the sensory and motor faculties, whereas human beings possess all of these, as well as the intellectual faculty. The distribution of the faculties of the soul infers a hierarchy between beings without, however, establishing an ontological break. There is continuity between beings.

For Aristotle, things possess a unique, necessary and universal essence. This point to an essentialist epistemological universe in which things are constant. The invariability of the essence of things implies that things, in their essence, pre-existed before human beings, and are, and will always be, what they are at the moment. Thus the essence of a thing is conferred by its “shape” and its function. In the teleological universe of Aristotle, there exists a narrow relationship between the shape of organs and their functions. The function determines the shape of the organ. As Wolff stated, “Metaphysical essentialism is linked to biological fixism” (2010, p. 166).^1^

The good, for man as for things, consists in being in compliance with one’s essence. Existing is to live according to one’s type, according to that which defines one. It appears that every thing has its natural place that is also its shape and its purpose. For Aristotle, human beings and living things encompass each other as one being that is part of a whole or of nature, insofar as it designates the totality of the being in movement. For example, in the case of the heart and the body, the relationship is one of the parts within the whole. It would be absurd to isolate the heart from the body. Without the body, the heart is no more than a heap of cells, and without the heart, the body becomes a corpse. Therefore, there are movements that may be called violent because they run counter to nature, insofar as these movements are influenced by an outside force. A heart is not of itself transplanted into another body. It requires outside forces.

And so, the art of curing derives its principles from the knowledge of nature. In this way, Aristotle distinguishes the art of curing understood as the efficient cause and health understood as final cause, as what strives towards its true shape or true nature (1941, p. 238). As for human beings, their highest function consists in contemplating nature. Human beings thus find in nature an immanent limit. Therefore, for the Greeks, there exists an intelligence specific to nature. It is in this sense that for Aristotle, the good *par excellence* consists in performing its function (1941, p. 942). Nature, as much for Aristotle as for the ancient Greeks, appears as a cosmic order that preserves itself and is renewed in eternal return (Gadamer 1995, p. 56). In this frame of mind, medical intervention consists in restoring a disrupted balance. It does nothing other than state the conditions for restoring the balance. Knowledge is limited to observing and discovering this natural intelligence. Thus it was a matter of doctors intervening as little as possible in order to respect the natural order. They were required to intervene, but remain mindful to act only to restore natural harmony. Transplantation is in no way natural. It stems from an outside, accidental and violent force.

Christianity brought the first upheaval of this idea of nature as a self-referential entity. Nature became a creation of God from which God excluded himself. This marked the “desubstantialization” of nature. The genius of Descartes, if indeed genius there were, would consist in claiming that we could explain nature without resorting to God, its creator.

## The point of view of Descartes

Descartes had an immense impact on the development of medicine. Although he was not himself a doctor, he was surrounded by many prominent physicians such as Vopiscus Plemp, Johan van Beverwijck, Cornelis van Hogenlande and Johan Elichman. In *Discourse on the Method*, published in 1637, Descartes expressed his interest in medicine. He wished “to acquire some knowledge of nature from which we may derive rules in medicine that are more reliable than those we have had up till now” (1985, p. 151). Medicine, based on the knowledge of nature, would make human beings wiser. Descartes again revealed his interest in medicine in a letter to Huygens in January 1638, “Je travaille maintenant à composer un *Abrégé de médecine*, que je tire en partie des livres et en partie de mes raisonnements” .^2^ Also in a letter to Mersenne dated February 20th, 1639, Descartes mentioned that he had been practicing dissection on animals for several years. “I have spent much time on dissection during the last 11 years, and I doubt whether there is any doctor who has made such detailed observations as I” (1991, p. 134). However, Descartes’ research on physiology was limited to the heart. Consequently, when he gave advice, in the absence of discovering “a medicine which is founded on infallible demonstrations” (1991, p. 17), he probably relied on Hippocrates and Galen (Lindeboom 1978, p. 42). Similarly, when he faced moral questions, Descartes relied on tradition. This could be termed clinical medicine by provision.

In order to succeed in basing medicine on indubitable knowledge, Descartes developed a method that consisted in separating mind from matter. This method may be defined as methodological dualism that allowed for a conception of the world according to two substances: *res extensa* that corresponds to the world of external bodies determined by extension and movement, and *res cogitans* that corresponds to mental substances. Whereas these two spheres of reality are separated on a methodological level, they are bound to each other in the human experience. This subject will be dealt with further, later on. Descartes believed that the body was a substance which contained nothing of the mind. Moreover, because animals do not possess thinking souls, they were perceived by Descartes as pure automatons.^3^ In fact, the body, such as presented in the sixth *Meditation on First Philosophy*, was seen as a machine. Descartes compared it more precisely to a clock or to a fountain. Thus, he demonstrated that bodies were subject to the action of natural and mechanical laws, and not to outside force, as suggested by Aristotle, who proposed a principle of animation that he believed resided in the soul.

For Descartes, the universe, “the earth as the heavens”, is made of a single matter in such a way as to occupy “all conceivable spaces” (1985, p. 232). In brief, “there is but a single matter existing in the universe” (1985, p. 232). It derives its own properties “from the movement of its parts” (1985, p. 232), so that we might say that the body is seen as a machine that generates its own movement much as do “clocks, artificial fountains, mills, and other such machines, that, although man-made, have the power to move under their own power” (1985, p. 99). All body movements arise in one way or another “without any contribution from our will”. For example, respiration, digestion, walking, sleep, for human beings as well as for animals, are actions that are driven “the same way as the movement of a watch is produced, simply by the strength of its spring and the configuration of its wheels” (1985, p. 335). *In fine*, in order to explain the mechanism of this machine, we do not need a vegetative or sensory soul or any other principle of movement and life” (1985, p. 108) such as clarified by Aristotle in his treatise *On the Soul*. Even the passions of the soul, which are thoughts, albeit thoughts that owe nothing to our will, are explained by the mechanics of the body.

The use of the machine metaphor to represent the body went on to govern modern western medicine in a decisive way. Even today, the practice of artificial organs, of organ transplantation and donation perfectly illustrate this image of a machine, as if it were only a question of changing parts to restart the machine.

However, Descartes did not reduce human beings to an assemblage of mechanical parts. He writes: “I am not that assembly of limbs that is called the human body” (1984, p. 18). While this model of the body, as a machine, possesses a most interesting heuristics function on the epistemological level, it is different on the moral level of everyday life. There is a discontinuity between the two. In others words, when the moment comes for human beings to determine their lives, they cannot rely solely on theoretical knowledge of what life is. They must take into account their experience of life which never appears perfectly clearly, but emerge more often in an uncertain and cluttered manner. Human beings, as opposed to animals that act without thought, according to Descartes, cannot make moral decision in a mechanical way, because they are spirits endowed with reason and will, and are conscious of inhabiting a body. What makes man a human being, is the union of body and soul (1985, p. 99). In this way, whereas nature as an object of thought is non-normative, it is different when nature is lived through the fusion of body and soul. If we return therefore to organ transplantation, it becomes not only a question of repairing the body-machine, but a life decision that asks the question: *Quod vitae sectabor iter?* (Which path of life shall I choose?).^4^

When the time comes to make decisions regarding the orientation of life, human beings do not rely solely on knowledge established by objectivity, but more on judgment that finds its source in subjective experience. For example, a disease often suffered in pain and entailing sadness, teaches us to deal with our finite nature due to the union of body and soul. In the sixth of his *Meditations on First Philosophy*, Descartes showed that the soul is not accommodated by the body “as a pilot in his ship” (1984, p. 56). Because of its embodiment in a body, the soul experiences sensations, appetites, and passions through which it feels its body not as a foreign object, but as its own body. The knowledge that human beings have of their body is not a conceptual knowledge, but an experienced knowledge that escapes the representations of the intellect. This is why Descartes thought that he knew “the animal in general […] and not yet man in particular” (1991, p. 134), because “those that belong to the union of the soul and the body understand each other only slightly by the intellect alone […] but very clearly through the senses” (1991, p. 226).

Within this knowledge acquired through experience, it is nature that enlightens human beings.

Nature also teaches me, by these sensations of pain, hunger, thirst, and so on, that I am not merely present in my body as a sailor is present in a ship, but that I am very closely joined and, as it were, intermingled with it, so that I and the body form a unit (1984, p. 56).

The experience of passion is not only the mechanical effect of the body, but above all the expression of the true man (1985, p. 141), that is the reasonable man, the one who makes use of his judgment. For Descartes passions are useful for the conservation of life (1991, p. 300), because “they dispose our soul to want the things that nature deems useful to us, and to persist in this volition” (1985, p. 349). In brief, in order to exercise his reasonable soul or his judgment, the human being must rely upon life (1991, p. 300) and refrain from using only the intellect.

When human beings are sick, they seek the good of the body. Then, for Descartes, “the goods of the body […] do not depend absolutely on us” (1991, p. 325). It is incumbent to rely solely on “the power of nature” (1991, p. 1280). Descartes returns to the concept of nature and its principle “*vis medicatrix naturae*” such as understood by the ancient Greeks and found in the Hippocratic tradition. This is the reason that he proposes therapeutic interventions to Princess Elizabeth aimed at resting the mind, because a rested mind is more receptive to the healing forces of nature, especially if the mind places itself in harmony with nature by observing it and dwelling within it. Thanks to the art of medicine, it is possible to prolong life provided that human beings draw their inspiration from animals or nature to regulate their lives (1991, p. 1402) insofar as nature “understands more clearly” than any doctor [what is needed] for its own restoration. Man must focus his attention on the intelligence of his own experience to guide him in his choices for a healthy life. In this way, a reasonable man is one who relies on his own life experiences to decide what is good for him, in this particular case, his health. In order to acquire this highly useful knowledge of life, Descartes holds that we must turn to practical rather than speculative philosophy (1985, p. 142). This philosophy which resides halfway between the clarity of knowledge and the confusion of the senses, is the morality that appeals to our judgment rather than to our knowledge and to our senses, despite of the attendant risks.

## The naturalist point of view

Regrettably, what the history of western thought retains of Descartes amounts to his methodological dualism. Descartes, a worthy disciple of Aristotle, in spite of the severe criticism he drafted with respect to Aristotelian epistemology, does not confound the order of knowledge or theory with the order of judgment or practice. Both rely on the understanding of nature to feed the judgment on life. However, with the development of new technologies at the end of the XXth century, confusion settled in between these two language games. According to Habermas, “the establishment of new technologies that deeply permeate substrates of the human person that used to be regarded as ‘natural’ promotes a naturalistic self-understanding among experiencing subjects in their interactions with one another” (2008, p. 239). Also confused is the order of causality with that of validity. What of this naturalism? It remains very difficult to give an exact definition of naturalism given its long history and its ramifications. For our purpose, two types of naturalism can be delineated: ontological naturalism and methodological naturalism. To simplify, let us say that ontological naturalism amounts to the idea that reality contains no supernatural entity and that methodological naturalism is a working hypothesis helping us to better understand nature which, in many cases, proves fruitful from a scientific point of view.

This naturalism, since Darwin, demonstrates that human beings are animals or living creatures much like other creatures having gone through a lengthy evolutionary process. Nothing distinguishes them from animals. Even mental phenomena, which have appeared until now as the privilege of human beings, are seen as natural phenomena. In short, human beings are natural beings (Schaeffer 2007). Whereas, for Descartes, thought resides outside of the body, naturalism suggests that thought is an integral part of nature.

The only question for human beings becomes that of their adaptability to their environment. In this respect, the issue of organ donation or transplantation is part of the conversation between the self and the species. In other words, the individual will no longer rely on the intelligible, but directly on nature.

This type of naturalism may be found in ecological movements and among animal defence groups. The various currents of animal ethics show that between human beings and other species, there exists only a difference of degree. This takes us back to the reflection of Montaigne on animals in his text *An apology for Raymond Sebond* (2003). In this way, the moral duty of human beings is to care for the community of living creatures. There is no longer any immortal soul, but only living creatures that must be protected. We have moved from a working hypothesis to a real definition of a human being. Otherwise stated, from the description of a human being, it is possible to conclude certain prescriptions. The sources of our thoughts become the reasons for our choices of action. It is no longer the *ego* that thinks, but the brain. This leads to an ontological monism according to which “reasons and causes are two aspects of a single phenomenon” to the extent that “reasons represent the subjective side –the ‘experiential form’- of neurologically observable processes” (Habermas 2008, p. 158). In doing so, we so naturalize both the morality and the contents of thought.

For Habermas, what “is so unsettling is the fact that the dividing line between the nature we *are* and the organic equipment we *give* ourselves is being blurred” (2003, p. 22) through the prowess of biotechnology. Biotechnological intervention replaces the relationship of care and breaks the link between nature and human beings. The “manipulation […] rescinds the distinction between clinical action and technical fabrication with respect to our own inner nature” (2003, p. 50). The individual cuts himself off from the intelligence of nature for the benefit of his preference: preserving his life. In light of such a perspective, it is easy to understand why organ transplantation becomes an undisputed imperative. With naturalism, everything is nature, but a nature divested of meaning.

## Phenomenological and hermeneutical perspectives

It is entirely possible that nature and disease possess no meaning. According to Spinoza, the movement of nature relies upon its own mechanical laws and not on intention. Rather, it is human beings who would attribute a meaning to these laws. This is why, for human beings, it is not merely about merely existing, but about being something connected to a world of meaning. There is a finality which consists in reaching a degree of superior achievement. From then on, in the world of experience, imbued with the desire for meaning, human beings remain Aristotelian. When the moment comes to deliberate, human beings do not rely only on theoretical (*theoria*) or technical (*technè*) knowledge, but on their judgment (*phronesis*) (Aristotle 1941, pp. 1026–1028) nourished by inter-subjectivity and culture. Aristotle and Descartes, contrary to the defenders of naturalism, understood correctly that when it comes to giving an orientation to life, it is necessary to rely on a further rationality based on a dimension of experience.

Phenomenologists, for their part, are interested in the body in action, seeing in this a source of wisdom. The body possesses the capacity of executing spontaneous movements. These movements are made possible by a native dynamism that dwells in the body. When human beings are not affected by disease, their movements deploy effortlessly, “naturally”. Human beings are carried by vital dynamics. They are engaged in a fundamental suffering (*patior*). In this way, Jürg Zutt declared that human beings were “carried” (*Getragensein*) by organs, physiological and psychic functions that provided rules for the future (1963). Hubertus Tellenbach, saw in this vital force of the body an appearance of “endogeneity” (1980). The concept of “endogeneity” expresses that which is deeper in human beings. Human beings undergo these vital processes. They rely on all the “non-volitive” (1980, p. 40). However, this suffering is not an absolute. It is something from which human beings enter into a dialogue. Paul Ricœur (1966, p. 275) underlined it well. “Human existence is like a dialogue with an involuntary multiple protean involuntary”. He detailed this experience in the following way: “I submit to the body that I guide” (1966, p. 276). However, human beings do not drive the body according to their good will. Quite the contrary, they move it according to the appropriate requirements of the body. There is thus circularity between the voluntary and the involuntary. This explains why to go against this native spontaneity is to break a kind of natural law. However, if this recognition of the body’s own intelligence, as witnessed in animals, can become a guide for human behaviour in the face of situations, in the here and now, the fact remains that this knowledge is useless in making decisions about fate, except in recognizing that organ transplantation can still grant human beings insight into the body.

It is difficult to clarify this natural intelligence, especially since our understanding of this intelligence is dependent on our culture, our history and our biography. It is important to exercise caution. The greatest caution comes from acting prudently or, according to Heidegger, in letting be (*Gelassenheit*). Consequently, while waiting for and hoping for a better certainty, human beings have to deliberate and rely on their own thinking. As Hegel pointed out, thought is not reducible to nature (1963, p. 32) nor is it a by-product. *A contrario*, thought gives meaning to nature. Thus, human beings, because of the thought they exercise, become the place where nature becomes aware of itself.

Given human finiteness, the meaning of existence never appears in its objectivity, but always according to human subjectivity. If human beings have no direct access to the meaning of nature to provide enlightenment in relation to their own lives, they can gain a better understanding if they rely on the story of their lives. The life story substitutes for nature in granting a better understanding of existence. This does not mean supplying explanations on our life in the case of events provoking other events. The story of a life emerges from our reading of these events in regard to an undefined finiteness: that towards which human beings go without knowing where they are going, but that nourishes thought.

From the moment human beings deliberate on possible life choices, they are struck by their own ignorance, and the uncertainty that accompanies it. According to Kant, in this moment of crisis, human beings leave behind the determinism of nature to rise to the world of freedom. This freedom does not consist in acting indiscriminately, but rather in making choices that relate to a life story in search of meaning that makes possible an outcome to the intrigue of personal life. This intrigue at the heart of our life stories revolves around the desire for self-fulfillment. Consequently, organ transplantation is not only decided by relying on arguments provided by the intellect, but on reading a life story that tries to understand and accomplish itself. That is why it becomes essential to reflect on the way the donor and receiver experience the organ donation.

## Donor and recipient experiences

A person could find devastating the idea that his or her life depends from now on on a gift from another person. The gift could have many meanings as much for the donor as for the recipient. As Fox and Swazey (2002) noted, organ transplantation is similar to the dynamics of gift exchange. The process involves giving, receiving and reciprocity. The *locus classicus* on the subject of gift giving is Marcel Mauss’s *The Gift: forms and functions of exchanges in archaic society* (1990) elaborated in the 1920s. According to Mauss, despite the common notion that gifts are voluntary, they are in fact obligatory. To receive a gift is to become “indebted” to the donor. The recipient becomes obliged to reciprocity. Fox and Swazey designate this experience as the “tyranny of the gift” (2002). This exchange model can explain “the strains and stresses that donors, receivers and their families often experience” about an organ transplantation (Gill and Lowes 2008; Conrad and Murray 1999).

For living kidney donors, the act of donation in helping another has been described as one of the most altruistic and meaningful acts. The donor experiences emotional, psychological and spiritual benefits. This consists mainly of an increased self-esteem (Franklin 1994; Gill and Lowes 2008; Brown et al. 2008). Also, it has been shown that the donor’s decision-making process was instantaneous and involved little deliberation (Gill and Lowes 2008). Helping to restore the health of another comes spontaneously. It is not something that is conceived as “heroic”. Rather it is a “natural thing to do” as the donor had “no choice” (Gill and Lowes 2008; Zeiler et al. 2010; Alnaes 2012). The donor does not expect any kind of return. The “joy of giving” is worthwhile in itself (Godbout and Caille 2000). An American study reported that the donors did not regret their donation (Pradel et al. 2003). Therefore, giving an organ has a transformative and positive impact on the donor such as a feeling of happiness (Simmons et al. 1987; Johnson et al. 1999; Fehrman-Ekholm et al. 2000; Burrough et al. 2003; Stothers et al. 2005).

But some potential donors refuse to donate for fear of adverse effects (Sajjad et al. 2007), the expectation of a possible kidney failure in the future, the concern of having done something in vain because the transplantation was not a success (Lunsford et al. 2007) or for religious and ethnic influences (Wakefield et al. 2011). Research has also drawn attention to various reasons for the refusal to donate on the part of families (Simonoff et al. 2001). Some reasons are related to the context of the death, the timing of the request, the place where the request is made and the person making the request. This is not to mention the possible discomfort of having to make a decision in place of the deceased person.

## The case of Jean-Luc Nancy

Recipients are characteristically very grateful to their donors (Gill and Lowes 2008), even though some recipients will refuse any offers of transplantation from family members to avoid any kind of indebtedness (Olbrisch et al. 2001).

The French philosopher Jean-Luc Nancy, himself a heart and lung transplant recipient, describes his story in a short book (2010). He shows that the experience of being a transplant recipient is an extreme almost unimaginable one and therefore difficult to communicate within our every day world. The kidney, the heart, the lungs are certainly corporeal, but they remain somewhat intangible like, according Derrida (2005), *Psychè*. Their near absence causes us to talk about her, *peri psyche,* as something strange and queer. In fact, like Nancy said, we do not recognize ourselves. The relation to the self is no longer apparent, immediate. In this sense, the recipient emerges lost from this experience. And the experience never stops because the intruder, the transplanted organ, instead of naturalizing itself, continues to intrude. Thus, it is necessary to receive this stranger and honour his presence. It becomes possible to say that we are no longer one, but rather many, so that the boundary between oneself and the other is not clear as is the frontier between death and life, young and old. Everything meshes. But the touch of another results in feeling oneself, that we touch ourselves, and that we are there and alive.

From a psychoanalytical perspective, people say that being a recipient is like having somebody inside as though the donor were there everyday. Even if the donor has died, he is still alive. Therefore, the organ is not a mechanical part, but a living organ. “What is inside me is a life” (Goetzmann 2004, p. 282). In this case, we cannot say that the technology that renders organ donation and transplantation possible dehumanizes the organ (Sharp 2001, p. 115).

What the recipient receives in transplantation is not merely an organ or a body part, but “life” itself (Deguchi 2002). The body we have is the first gift of life, in the sense that it is not only a utilitarian object, but that by which we receive life. The body, in addition to having its own history, is the site of another story. It serves as a condition to live in closer harmony with the meaning we give to a good life. It is not an end in itself, but a means. Both giving and receiving a body is to give meaning to life.

## Caring and thinking

Illness teaches us that there is a gap between what we are at present and what we could become. Organ transplantation would close this gap. Nevertheless, in a certain sense, it is precisely this gap between the meaning that we give to nature and the withdrawal of nature in regard to this meaning that makes human beings alive by constantly relaunching the dialogue with nature on new terms. What is at stake in organ transplantation is how the truth plays out.

Living with a new organ goes beyond the simple fact of being still alive. The recipient must elaborate anew his relationship to the world: a precarious life characterized by a lot of mourning. This implies a search for a new wisdom to transform illness into a initiatory journey that flies in the face of social convention and finds what is essential to existence.

William James in *The Variety of Religious Experience* insisted on the spiritual value of an encounter with death. In the eyes of Jaspers, facing death becomes a limit-situation. As with all limit situations, the human being is confronted by his own incomprehension. Can it be said that a person who receives organ transplants misses an opportunity to experience an awakening to the human condition through the mourning of both certain desires and a part of themselves? The fact of tying a medical and psychological answer, that is to say technical, to existential questions distracts human beings from reflection. This attitude cannot but create a new existential blockage (Jaspers 1966, p. 92). *A contrario*, again according to Jaspers, reflection upon the sense of human existence presents itself as the place where we are ourselves (1966, p. 94) that which places us on the road to comprehension, without however claiming certainty. In this sense reflexivity is at the very core of the human condition. Within it is bound up the relationship with oneself, with others and with the mystery of existence. It constitutes the work of living: learning from our own life in order to live better (Montaigne 2003, p. 425). Being confronted with one’s own death becomes an occasion to mature, to awaken to oneself. Thus, what keeps a human being alive cannot be counted merely in terms of medical interventions, but rather in the care taken in reflecting on the sense of existence in relation to his own accomplishment.

We have indicated that reflecting on the sense of existence does not necessarily lead to certainty. It would be a mistake however to believe that arbitrariness is acceptable. In order to avoid the arbitrary, it is important to carry out the reflection in a context of intersubjectivity constituted by the Socratic dialogue (Nelson 1965). We need others to nourish our reflection. In this respect, Socrates is surely an exemplary figure. He believed, according to Plato’s *The Apologie of Socrates* (38a), that a life worth living is one which examines itself. Without offering any solution or knowledge, the role of the philosopher in raising relevant questions consisted in helping other people to give birth to their own ideas. This is the reason why Socrates or the philosopher is described in the Plato’s *Theaetetus* (150c–151b) as a midwife.

In this sense, philosophy does not consist in manipulating abstract concepts, but in receiving “philosophical questions posed by life” (Lahav and da Venza Tillmanns 1995, p. x). That is why the “entire field of medical ethics, for instance, with its dilemmas regarding euthanasia, abortion, extraordinary means of prolonging life, etc. is necessarily part of philosophical counselling” (Mijuskovic 1995, p. 92). But it is not only the medical field that offers opportunities to think about life. In fact, life itself is “a continuous interpretation of ourselves and the world” (Lahav 1995, p. 24). Charles Taylor (1985) designates human beings as self-interpreting animals. According to Achenbach (1984), this concerns philosophical questions issuing from life and is the subject matter of philosophical counselling.

From that moment on, “philosophical counselling offers a controlled and directed environment in which life—herein understood as a process of interpretation- is intensified” (Lavah 1995, p. 24). It consists in rendering explicit what is hidden or implicit. This act of hermeneutics enables a better phenomenology and this improved phenomenology, in return, intensified the act of hermeneutics.

Thus, organ transplantation can become a final appeal that precisely allows the prolonging of life and the multiplying of opportunities to experience it, because after death, there is no longer any possibility of experiencing. Heidegger in *Being and Time* explained that with death comes the possibility of the impossible. The body thus deserves quality care. Of course there is always the chance of being reduced to an animal condition in a world deprived of meaning.

The body serves as a support to the experience of awakening which in turn enlightens nature and our life stories by giving them meaning, i.e. a reason for being. In this respect, awakening gives access to different and immeasurable levels of reality: nature and meaning. Thus, if we rely on the Greek meaning of the verb to meditate, *medow*, to meditate on nature and to care for nature as one would one’s body constitutes the same undertaking.

The first care we can bestow upon the body consists in thinking it. To think it is to act on nature that, in turn, acts on thought. It is as though by thinking nature, human beings give themselves their own determination. The important point consists in establishing a hermeneutic circle between what we think and what reminds us to think. In this way, the experience of nature is always mediated by the language of culture understood as the repository of questions and meaning. In other words, meaning gives access to nature while simultaneously being an event of nature. Within this intelligible order, nature encounters itself.

In the *Odyssey*, Ulysses, then a prisoner of Calypso, is offered immortality if he stays by her side. Ulysses refuses the offer, because he wishes to remain a human being, that is, a mortal. Therefore, human beings deliberate and make choices in consideration of the way they understand themselves and their lives. The most important thing is the occurrence of understanding. For that to happen, human nature must live, but live a life that accepts not to understand everything. What makes human beings worth living is the desire for self-understanding in relation to the fulfilment of self. In other words, it is the act of thinking and of caring that gives meaning to human existence.

## Conclusion

If today organ transplantation is a common place occurrence, it is no less true that the subjective experience of it in relation to the good life may remain unintelligible. We have attempted to understand through an examination of Aristotle, Descartes and naturalism, that human beings have relied on an interpretation of nature to gain greater understanding and thereby help orient their lives. Finally, the story of Nancy and others shows that it is in the experience itself constituted by interpretation, as much for the donor and the recipient that it is possible to better understand what is entailed and to give it meaning. The human experience of organ transplantation demonstrates that thinking about and caring for the body is part of the same undertaking in the sense that it is the act of thinking as well as caring that gives meaning to human existence. For that to happen, we need the presence of others inside a Socratic dialogue as we encounter it inside philosophical counselling. The reflection on the good life is prescribed by our own essence: that of the self-interpreting being. The possibility of organ transplantation depends a great deal on the culture we live in: a culture of interpretation. Organ transplantation becomes an extreme experience that intensifies philosophical questionings, a hermeneutical stance about oneself and the world.

## Footnotes

1 My own translation.

2 Lettre du 25 janvier 1638, Ch. Adam et P. Tannery, *Œuvres de Descartes*, I, 507 tiré de G.A. Lindeboom, *Descartes and Medicine*, Amsterdam, Rodopoi, 1978, p. 35. “I am working now on composing a *Summary of Medicine* that I am deriving partially from books and partially from my reasoning” . My own translation.

3 Descartes stand in opposition to the vision of Montaigne (Cf Essays, II, 12) according to which the difference between human being and animal is a difference of degree, whereas for Descartes, it is about an ontological difference. Cf *Discourse on the Method*, (1985, p. 140).

4 This question was revealed to Descartes in one of his dreams on the night of November 10–11 1619.
